# Simultaneous Hydrogen Production and Dye Decomposition in Alkaline Photocatalytic Process Using Calcined Xerogels of CuO-TiO_2_

**DOI:** 10.3390/gels11050319

**Published:** 2025-04-25

**Authors:** Susana López-Ayala, Elsa C. Menchaca Campos, Miguel A. Méndez-Rojas, Marina E. Rincón

**Affiliations:** 1 Centro de Investigación en Ingeniería y Ciencias Aplicadas, Universidad Autónoma del Estado de Morelos, Avenida Universidad 1001, Chamilpa, Cuernavaca 62209, Mexico; 2 Departamento de Ciencias Químico-Biológicas, Escuela de Ciencias, Universidad de las Américas Puebla, Ex Hda. de Santa Catarina Mártir s/n, San Andrés Cholula, Puebla 72820, Mexico; 3 Instituto de Energías Renovables, Universidad Nacional Autónoma de México, Privada Xochicalco s/n, Temixco 62588, Mexico; merg@ier.unam.mx

**Keywords:** photocatalysis, system CuO-TiO_2_/AB1/NaOH, AB1 discoloration, hydrogen

## Abstract

Research on hydrogen (H_2_) production has been intensively investigated due to the critical need for transitioning from fossil fuels to cleaner energy sources. This study demonstrates a dual-purpose approach where water pollutant degradation and H_2_ production occur simultaneously, eliminating the need for sacrificial materials and reducing costs. CuO-TiO_2_ calcined xerogels were employed in solutions containing NaOH and acid black dye 1 (AB1). The CuO-TiO_2_/AB1/NaOH system successfully degraded recalcitrant pollutants while producing H_2_ under optimized conditions. H_2_ evolution occurred at the photocatalyst holes due to AB1’s lower potential compared to water, while AB1 decomposition proceeded via O2^•−^ radical formation. X-ray diffraction (XRD) and Scanning Electron Microscope (SEM) analyses showed sponge-like structures with 20 nm crystals. Polarization curves confirmed H_2_ generation in the cathodic region. Bode diagrams of the CuO-TiO_2_/AB1/NaOH system (0.3 M NaOH and 60 mg/L AB1) exhibited noble/passive behavior, consistent with the polarization curve data. Using 0.3–0.4 M NaOH and 60 mg/L AB1, 636–647 ppb H_2_/g_catalyst_ was produced in 60 min, and only 0.07 mg/L AB1 was left as indicated by absorbance measurements at 618 nm. H_2_ evolution decreased as dye degradation increased. The best system for dye degradation has a k constant of 0.066 min^−1^ and R^2^ of 0.99, contains 40 mg/L AB1, and runs at 40 °C, whereas the maximum dual performance required 0.5 M NaOH, yielding 5050 ppb H_2_/g_catalyst_.

## 1. Introduction

**Figure 1:**
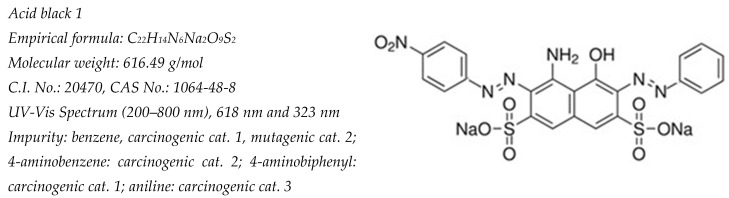
Characteristics and chemical structure of acid black dye1 [14]. Adapted from European Commission: Directorate-General for Health and Consumers, Opinion on Acid Black 1 COLIPA n° B15, European Commission, 2010. https://op.europa.eu/en/publication-detail/-/publication/8022fd75-43d5-4542-b90c-6266a287cf55/language-en. Accessed on 1 September 2024.

Since the industrial revolution, large-scale fossil fuel consumption has prompted growing concerns about environmental pollution, associated health problems, and climate change [1,2]. This has accelerated the transition to carbon-free energy sources [1,2,3,4]. Hydrogen (H_2_) has emerged as one of the most promising clean energy carriers, with active research exploring its technological applications [5,6]. Common H_2_ production methods include electrolysis, photocatalysis, and Photoelectrocatalysis, typically using (NaOH) or acidic (H_2_SO_4_) aqueous media, often with sacrificial compounds such as Na_2_SO_4_, TEA, alcohols, and organic compounds [3,7]. Notably, dyes have been employed in coupled photocatalytic reactions that simultaneously produce H_2_ and degrade pollutants [8]. Recent years have seen significant efforts to develop photocatalysts capable of both H_2_ generation and water remediation [8,9,10,11]. Such systems require strong adsorption of the dye on the photocatalyst, appropriate alignment of the energy levels, and compatible redox potentials for both hole-driven dye degradation and electron-driven hydrogen production. Synthetic dyes represent particularly problematic recalcitrant organic compounds in aquatic environments [12]. These compounds are extensively used across various industries, including dye manufacturing, textiles, food processing, paper production, plastics, and printing. Approximately 10–15% of total dye production enters the environment untreated [12], presenting an opportunity for coupled photocatalytic H_2_ production and dye degradation processes [13]. Acid black dye 1 (Figure 1) contains two azo (diazoic) groups that function as chromophore centers, enabling it to sensitize appropriate photocatalysts [14].

Photocatalysis can operate in both acidic or alkaline conditions, though alkaline environments are preferred for sustained large-scale hydrogen production. Consequently, the hydrogen evolution reaction (HER) is predominantly conducted under alkaline conditions in industrial settings, where long-term durability is essential [15]. Additionally, most photocatalysts demonstrate superior efficiency for HER in alkaline media [16]. The development of efficient and economic catalysts remains a critical challenge. While noble metals such as platinum offer excellent catalytic activity, their high costs limit their widespread implementation. Significant research has focused on developing alternative non-precious metal catalysts, including various metal oxides such as copper oxides and TiO_2_ [17].

Sacrificial agents are employed to consume holes generated during photocatalytic water splitting, though many are considered environmentally toxic [18]. These agents, with their less positive oxidation potentials, compete more efficiently for the photogenerated holes than the water reaction [18], avoiding electron/hole recombination and leading to a larger electron density at the heterodoped TiO_2_ surface available for photoreduction. The photocatalytic production of H_2_ with simultaneous dye degradation involves three key steps: (i) light absorption, (ii) the generation and migration of electron-hole pairs, and (iii) surface redox reactions. The distinctive feature of this process is that steps (i) and (ii) predominantly occur at the dye or dye/photocatalyst interface [9,18]. Energy level alignment plays a crucial role: the LUMO of the excited dye must be less negative than the photocatalyst’s conduction band, which in turn must be less negative than the Fermi energy required for H_2_ production. Concurrently, photogenerated holes may originate from the HOMO of the excited dye. Additional redox reactions can occur through photogenerated carriers, forming superoxide radicals (O_2_^•−^). These processes depend on the relative redox potential and photocatalyst band positions [7,9,19].

In alkaline photocatalytic reactions, the oxidation–reduction potential (ORP) is an important parameter that indicates whether an oxidation or reduction reaction is occurring. Generally, a positive ORP value indicates an oxidation reaction, while a negative value signifies a reduction reaction [3]. In our experience with photoelectrocatalysis under alkaline conditions, establishing a negative potential is crucial, although the ORP does not follow a consistent pattern or decline during the reaction.

### Recent Advances in Simultaneous H_2_ Evolution and Contaminant Degradation

W. Ding et al., from 2023 **[8]** discuss key advancements in photocatalysis and focus on the potential of ultrathin 2D ZnGa-borate-LDH nanosheets for enhancing photocatalytic reactions for both hydrogen production and pollutant degradation: ultrathin 2D ZnGa-borate-LDH nanosheets can boost dye-sensitized photocatalytic reactions, coupling H_2_ production with pollutant degradation.

In the study conducted by Wongyongnoi et al. (2024) [10], the focus was on the utilization of gold-decorated titanium dioxide (TiO_2_) as a photocatalyst to facilitate the simultaneous generation of H_2_ and the decolorization of distillery effluent. The photocatalytic process harnesses solar energy to drive chemical reactions, thereby promoting a green synthesis pathway that minimizes the reliance on fossil fuels and reduces the environmental impact of wastewater discharge. The results demonstrate a significant enhancement in hydrogen production rates and effective decolorization of the effluent.

L. Yang et al., 2021 [11] discuss enhancing photocatalytic hydrogen production along with dye degradation using Ni_2_P-modified In_2_O_3_ nanocomposites. Nickel phosphide might be acting as a cocatalyst, which can enhance photocatalytic activity by providing active sites or facilitating charge separation. The nanocomposite structure likely improves the surface area and interaction between the components, leading to better performance. In conclusion, the nanocomposite structure likely improves light absorption, charge transfer, and surface reactivity, enabling simultaneous H_2_ generation and dye breakdown.

Q. Wang et al. (2023) [20] and (2024) [21] used the Z-scheme to synthesize Sn_3_O_4_/TiO_2_ and Bi_2_O_3_/CeO_2_ as photocatalysts of Sn_3_O_4_/TiO_2_ and Bi_2_O_3_/CeO_2_/TiO_2_ nanotube arrays (TNTAs), respectively, with enhanced performance for dye degradation and hydrogen production. They synthesized Sn_3_O_4_ and Bi_2_O_3_/CeO_2_ nanoparticles grown on vertically aligned TiO_2_ nanotube arrays via a controlled hydrothermal process, to ensure uniform dispersion and strong interfacial contact between Sn_3_O_4_ and TiO_2_, which are critical for charge transfer. The heterojunction ensures that the composites form a Z-scheme charge-transfer mechanism, which enhances electron-hole separation and extends light absorption into the visible range. The TiO_2_ NTs provide a high surface area, directional charge transport pathways, and improved light-harvesting efficiency. With Bi_2_O_3_/Sn_3_O_4_/TiO_2_, (a) the composites exhibit superior degradation efficiency for organic dyes (e.g., RhB) under visible light due to enhanced reactive oxygen species (ROS) generation and (b) hydrogen production in the Z-scheme system promotes efficient water splitting, achieving higher H_2_ evolution rates compared to pure TiO_2_ or Sn_3_O_4_. For the optimized Bi_2_O_3_/CeO_2_/TiO_2_ NTs, it was demonstrated that they had (a) enhanced dye degradation efficiency (e.g., methylene blue) under visible light and (b) increased hydrogen evolution rates due to preserved redox potentials.

Q. Wang et al. (2022) [22] developed Bi-assisted modified CdS/TiO_2_ nanotube arrays (NTAs) with a ternary S-scheme heterojunction for simultaneous applications in photocatalytic wastewater treatment and hydrogen production. The system integrates TiO_2_ NTAs as a base structure, CdS nanoparticles as a visible-light-responsive semiconductor, and Bi species (likely Bi_2_S_3_ or metallic Bi) as a cocatalyst. The TiO_2_ NTAs provide a high surface area and ordered charged transport pathways, while CdS extends the light absorption to the visible spectrum. Bi modification enhances the charge separation and active sites. The ternary system forms an S-scheme charge-transfer pathway between TiO_2_, CdS, and Bi. There is an efficient degradation of organic pollutants (e.g., Rhodamine B) due to enhanced ^•^OH and ^•^O_2_^−^ radical generation from the S-scheme mechanism. The optimized heterojunction achieves higher H_2_ evolution rates compared to binary systems (e.g., CdS/TiO_2_), attributed to the improved charge separation and Bi’s catalytic role.

J. Liu et al., 2020 [13], discuss a photocatalyst composite film using Ag, AgBr, and TiO_2_ in a Z-scheme setup for simultaneously degrading malachite green and producing hydrogen. The composite film probably has a structure where Ag nanoparticles are between AgBr and TiO_2_ to facilitate electron transfer, leading to better performance in both degrading organic pollutants and generating hydrogen.

Z. Yan et al., in 2023 [9], discuss the role of photocatalysis in combining water remediation with hydrogen production. The review highlights the techniques for efficient photocatalytic processes, explores the environmental benefits of integrating these methods, and reviews various materials used in photocatalysis and their effectiveness with emphasis on synthesizing new photocatalytic materials that enhance efficiency and selectivity; their work also reviews the optimization of reaction conditions; discusses the importance of tuning parameters such as light intensity, wavelength, and reaction medium to maximize performance; and describes the coupling of photocatalytic and photovoltaic systems. It is clear that by fostering technologies that utilize renewable resources for energy and waste treatment, it is possible to achieve sustainable energy solutions and the overall health of the environment.

Regarding the use of CuO as a cocatalyst, M. Benedet et al., 2024 [17], reported the use of Cu_x_O–gCN–TiO_2_–Au (x = 1, 2) nanoarchitectures and demonstrated efficient photoactivated hydrogen evolution through water splitting, leveraging synergistic interactions among their components. A scalable synthetic route integrates copper oxides (CuO/Cu_2_O), graphitic carbon nitride (gCN), TiO_2_, and Au nanoparticles. The hybrid structure creates heterojunctions that enhance charge separation and light absorption. High hydrogen evolution rates are achieved under visible light due to Au nanoparticles acting as electron sinks and plasmonic enhancers. The system operates efficiently with or without an applied bias, indicating strong intrinsic charge transfer mechanisms. Cu_x_O facilitates hole scavenging and stabilizes charge carriers. gCN provides a conductive matrix and active sites for proton reduction. The advantages of this system are a synthesis that minimizes hazardous reagents and a fabrication method that is compatible with industrial applications. With respect to CuO-TiO_2_ photocatalysts, Méndez Medrano et al. 2020 [23] reports the heterojunction of CuO nanoclusters with TiO_2_ and demonstrates dual functionality in photo-oxidation of organic compounds and hydrogen production through enhanced visible light photocatalytic activity. The CuO/TiO_2_ heterojunction improves charge separation and extends light absorption to the visible spectrum due to CuO’s narrow bandgap. Under visible light, CuO acts as a photosensitizer, injecting electrons into TiO_2_’s conduction band, while holes remain in CuO. Although the degradation and evolution of hydrogen is not simultaneous, the system effectively degrades pollutants such as methylene blue and generates H_2_ with methanol as a sacrificial agent under visible light.

In this work, we used calcined CuO-TiO_2_ xerogels obtained by sol-gel synthesis and AB1 dye in alkaline media under environmental conditions without sacrificial agents. To monitor the reactions, we selected the oxidation-reduction potential (ORP), which was easy to implement. The ORP can decrease during H_2_ production when reduction conditions exist, such as with the use of NaOH and certain dyes containing reducing groups that can be coupled with the photocatalyst. Our data indicates that the CuO-TiO_2_/AB1 system can be optimized under alkaline conditions to simultaneously achieve H_2_ generation and AB1 degradation without the need of sacrificial agents.

## 2. Results and Discussion

**Figure 2:**
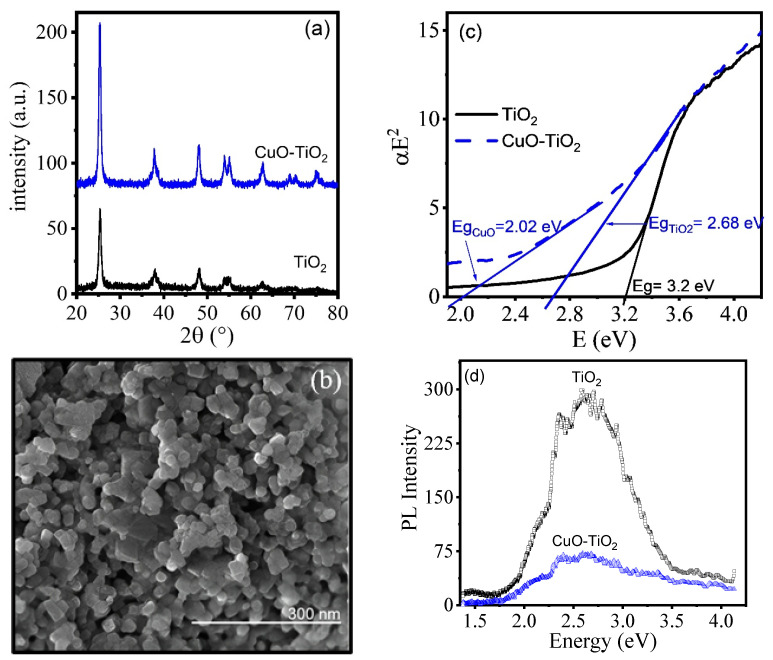
Characterization of TiO_2_ and CuO-TiO_2_. (**a**) XRD, (**b**) SEM, (**c**) band gaps obtained from transmittance data assuming direct optical transitions, (**d**) photoluminescence, PL.

XRD and SEM data of the calcined CuO-TiO_2_ xerogels are presented in Figure 2.

Figure 2a shows the anatase phase of TiO_2_ (JCPDS 21-1272) in both the TiO_2_ and CuO-TiO_2_ xerogels. No CuO peaks are evident, indicating either a good nanoparticle dispersion or heterodoping of the titania matrix. The crystallite size calculated from the (101) TiO_2_ plane is in the range of 19–21 nm for both CuO-TiO_2_ and undoped TiO_2_. The SEM images of CuO-TiO_2_ (Figure 2b) show sponge-like conglomerates with nanometric dimensions and no evidence of segregation into two different oxides (i.e., Cu_x_O and TiO_2_). The optical properties are shown in Figure 2c. Bandgap (Eg) determination was performed assuming direct transitions [24]. Extrapolation of the linear region gives approximately 3.2 eV for undoped TiO_2_. For CuO-TiO_2_, two linear regions were observed: the larger value at 2.64 eV corresponds to the red shift of anatase due to interband states, whereas the extrapolated value at ~2 eV can be correlated to the Eg of CuO, which has been reported in the range of 1 to 2.1 eV [25]. In the PL test, Figure 2d shows a larger PL intensity for TiO_2_ than for CuO-TiO_2_. Since light is emitted when electrons return to the ground state, the greater light emission from TiO_2_ indicates that the addition of CuO to the system prevents charge recombination due to the good coupling of the rectifying heterojunction.

**Figure 3:**
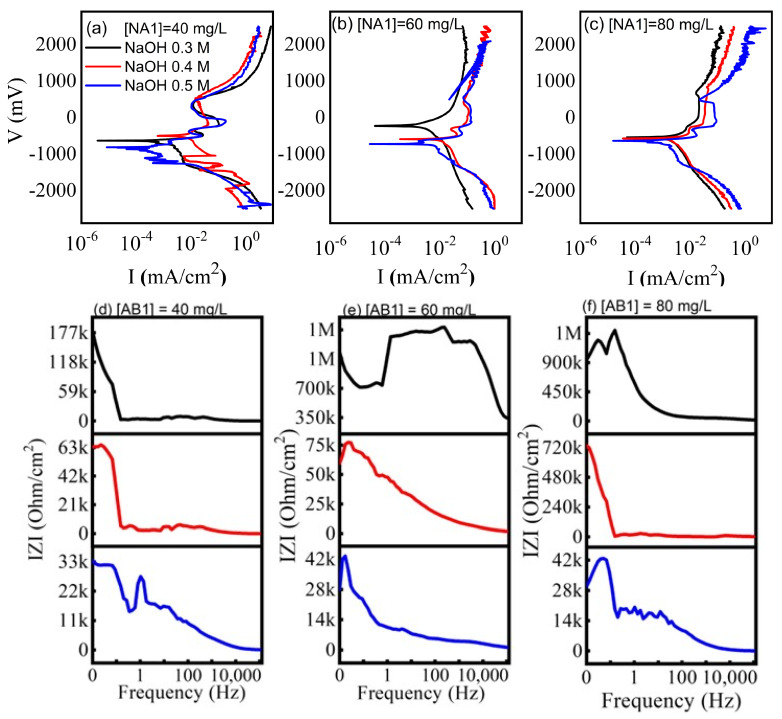
Electrochemical tests of CuO-TiO_2_/AB1/NaOH system as a function of AB1 and NaOH concentrations: (**a**–**c**) polarization curves, (**d**–**f**) Bode diagrams.

**Figure 4:**
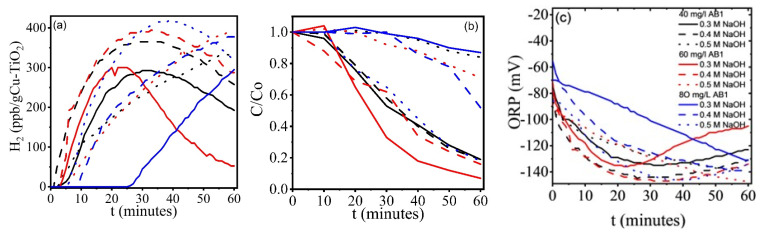
Simultaneous processes occurring in CuO-TiO_2_/AB1/NaOH: (**a**) hydrogen evolution, (**b**) AB1 degradation, and (**c**) ORP evolution. Tests performed under environmental conditions and visible light (laboratory light). AB1 concentrations of 40, 60, and 80 mg/L, and alkalinity of 0.3, 0.4, and 0.5 M NaOH.

**Figure 5:**
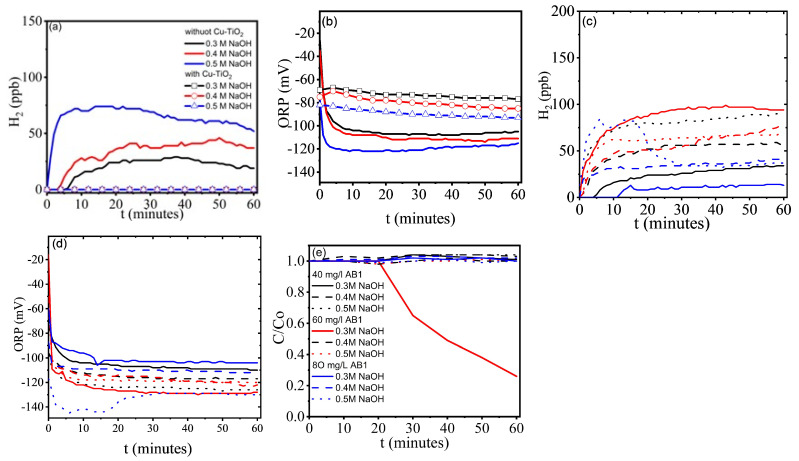
Blank tests under visible light (laboratory light) and environmental conditions. (**a**,**b**) Hydrogen evolution and ORP, Without AB1, with and without CuO-TiO_2_ xerogels; (**c**–**e**) photocatalytic hydrogen evolution, ORP, and AB1 Photodegradation without CuO-TiO_2_. xerogels, in basic media and 40, 60, and 80 mg/L AB1.

With respect to electrochemical studies, Figure 3 shows the polarization curves (Figure 3a–c) of the CuO-TiO_2_/AB1/NaOH system. The voltage applied ranged from −2500 to 2500 mV. Higher dye concentrations shift the curves toward more positive current intensities. Tests with 0.3 M NaOH and 40 and 60 mg/L AB1 showed more positive potential, with the combination of 0.3 M NaOH and 60 mg/L being the most passive and closest to zero (although still negative). The current oscillations observed in both the anodic and cathodic regions are due to oxygen and H_2_ evolution. From the electrochemical impedance tests of CuO-TiO_2_/AB1/NaOH, Bode diagrams were obtained at frequencies from 0.01 to 125,000 Hz (Figure 3d–f). The tests were performed in alkaline media with NaOH from 0.3 to 0.5 M and at various AB1 concentrations (Figure 3d), 60 mg/L (Figure 3e), and 80 mg/L (Figure 3f). The Bode diagrams show different behaviors depending on the dye concentration and alkalinity. The high frequency impedance is low in all cases except in experiments with 0.3 M NaOH and 60 mg/L AB1, where it reaches 350 kΩ. This high value was reproducible and may relate to the dye degradation process, which occurs more quickly under these conditions (Figure 4c and Figure 5e). The total impedance values increase with dye concentration and decrease with NaOH molarity, reaching its highest value at 0.3 M NaOH and 60 mg/L AB1, consistent with the findings of the polarization curves (i.e., more passive behavior).

### 2.1. Hydrogen Evolution and Degradation of AB1

Figure 4 shows the results of dissolved H_2_, ORP, and dye degradation obtained with the alkaline CuO-TiO_2_/AB1 system under the conditions described in Table 1. H_2_ production is favored at the highest alkalinity (0.5 M NaOH) and AB1 concentration (80 mg/L) (Figure 4a), consistent with the polarization curves. At 80 mg/L AB1 concentration, there is a delay when the solution alkalinity is low. Most curves show the maximum value expected when competing processes are occurring. This maximum is not observed at very high dye concentrations with low alkalinity, or at medium dye concentrations with high alkalinity. This correlates with the degradation curves shown in Figure 4b, where the experimental parameters causing a maximum in the H_2_ evolution curves also show the fastest dye degradation. In all cases, both dye degradation and H_2_ production occur. Figure 4c shows the ORP values; since they are negative, the system is apparently dominated by reduction reactions. Nevertheless, only those curves showing a minimum ORP can be correlated with H_2_ production.

Blanks were also conducted in alkaline solutions and alkaline dye solutions, with and without the CuO-TiO_2_ catalyst. Figure 5b shows the dissolved H_2_ and ORP values obtained for the alkaline solutions with and without the catalyst. ORP values more negative than −100 mV can be correlated with H_2_ production, and the data clearly show that CuO-TiO_2_ films inhibit H_2_ evolution. In alkaline AB1 solutions without CuO-TiO_2_ (Figure 5c,d), the AB1 concentration can be adjusted to achieve nearly 100 ppb dissolved H_2_. The effect of alkalinity depends on the AB1 concentration; at 40 and 80 mg/L, H_2_ increases in more alkaline media. Interestingly, at 60 mg/L AB1, the highest and steadiest H_2_ evolution was observed at the lowest alkalinity (0.3 M NaOH). ORP values more negative than −100 mV correlate with the H_2_ concentration. In the blank tests, the presence of a maximum (Figure 5c) or minimum (Figure 5d) is only observed for the highest dye concentration and maximum alkalinity, but contrary to Figure 4, AB1 degradation was not observed under these conditions. The simultaneous hydrogen evolution and dye degradation observed at 60 mg/L AB1 and 0.3 M NaOH does not correspond to the most negative values in the ORP curves, because the O_2_ in the environment accelerates degradation, but the evolution of H_2_ is not the highest.

**Figure 6:**
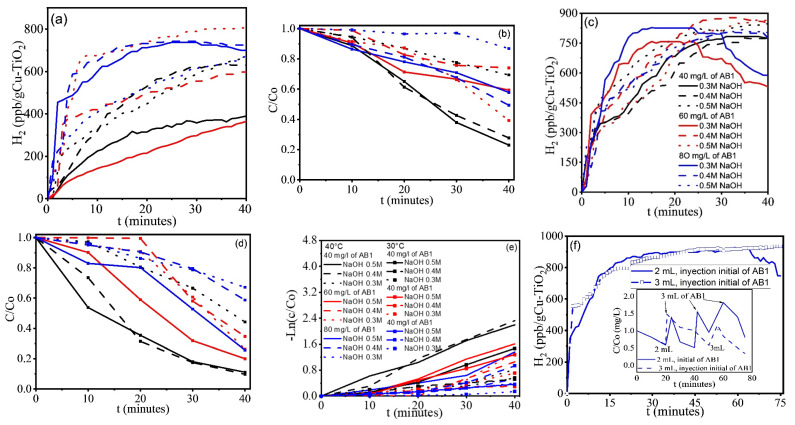
Hydrogen production and degradation of AB1with CuO-TiO_2_/AB1/NaOH systems: (**a**) H_2_ production at 30 °C, (**b**) AB1 degradation, 30 °C, (**c**) H_2_ production, 40 °C, (**d**) AB1 degradation, 40 °C, (**e**) Kinetics of AB1 degradation with the tested systems, and (**f**) initial injection of AB1, system 0.5 M NaOH/40 mg/L AB1, with periodic additions of concentrated dye. Controlled temperature, and visible light (laboratory light).

Temperature affects the performance of the sensors used to monitor H_2_ evolution. Although the ORP continues to decrease during the reaction, at temperatures between 20 and 23 °C (at the beginning of the experiments in Figure 6), H_2_ production decreases from 90 to almost zero (unconcluded experiment), while discoloration increases. The ambient temperature during the experiments in Figure 4 and Figure 5 was between 26 and 28 °C, and no measurement problems were encountered. Only when the ambient temperature dropped did H_2_ production decrease and AB1 degradation accelerate.

Figure 6 shows the results of experiments conducted at 30 and 40 °C (Figure 6a–f) in the alkaline CuO-TiO_2_/AB1/NaOH system, under controlled temperature and in a better sealed, though not airtight, reactor. For H_2_ evolution (Figure 6a,c), the values are approximately twice those reported in Figure 4, and at 40 min, the spread of values due to different experimental conditions becomes narrower at higher temperature. The observation that the highest H_2_ evolution occurs at the highest dye concentration with medium alkalinity, or at the highest alkalinity with medium concentration, remains consistent. The maximum value reached was in the range of 802–870 ppb/(g CuO-TiO_2_).

Another difference between the temperature-controlled experiments and those conducted under environmental conditions is the steady evolution of H_2_ for longer periods. Figure 6b,d shows the results of AB1 discoloration occurring simultaneously with H_2_ production at 30 and 40 °C. We observe that at 30 °C, lower discoloration is obtained with less dispersion across experiments (varying in dye concentration and NaOH) compared to what was obtained at 40 °C. This trend is contrary to what was observed for H_2_ evolution. Figure 6e shows the results of the AB1 discoloration kinetics (λ = 618 nm). Only seven systems exhibited first-order kinetics (Table 2). The initial discoloration was slow in systems that were not first order, but at longer reaction times, the kinetics accelerated as the ORP increased with reaction time, affecting the discoloration rate. Among all CuO-TiO_2_/AB1/NaOH systems tested, only those with 0.4 M NaOH and 40 mg/L AB1 at 40 °C demonstrated faster kinetics, with a rate constant of 0.066 and an R^2^ of 1. It was followed by the system with 0.5 M NaOH and 40 mg/L AB1 at 40 °C with k of 0.055 min^−1^ and R^2^ of 0.99 (Table 2). Figure 6f shows the results of experiments carried out at 40 °C, 0.5 M NaOH, and 40 mg/L AB1, with periodic additions of concentrated dye (1000 mg/L) to compensate for the AB1 degradation. In the first test, 2 mL of AB1 was added at 24 min, 3 mL at 42 min, and 3 mL at 51 min. 

In the second test, 3 mL of AB1 was added at 21 min and 3 mL at 56 min. Tests showed an increase in H_2_ up to 1927 ppb/g_CuO-TiO2_/h when 2 mL of AB1 was added initially, and 5050 ppb/g_CuO-TiO2_/h when 3 mL of AB1 was added initially (Table 3). H_2_ production was carried out under the conditions shown in the insert of Figure 6f. The results indicate that the equilibrium between hydrogen production and dye degradation is sensitive to changes in dye concentration, which can negatively impact H_2_ production. 

### 2.2. Discussion

From the characterization data, it was demonstrated that a facile and low-cost sol-gel synthesis can provide a CuO-TiO_2_ semiconductor heterojunction with the rectifying properties to minimize electron/hole recombination under illumination. Moreover, when combined with AB1 in alkaline media, photosensitization is enhanced, along with H_2_ evolution, since the excited dye can also funnel electrons to the photocatalyst. Apparently, dye oxidation is enough to remove the photogenerated holes and increase the electron density at the photocatalyst surface without the need for sacrificial agents. There is indeed a delicate equilibrium for the dual function to proceed simultaneously, and the optimum conditions must be found. At the start of degradation, the blue coloration of AB1 is reduced, and a lighter blue coloration emerges first, then turns violet, and finally red (red remains until the end of the reaction, resulting in the partial decomposition of AB1 in all experiments). At controlled reaction temperatures of 30–40 °C, the red coloration also decreases. However, at higher temperatures, degradation increases, and the red coloration becomes more evident.

From the data, we can discern the following:

- There are several routes for dye degradation, with the non-selective degradation by hydroxyl radicals being the most dominant in alkaline media.
- At temperatures greater than 40 °C, and even at 40 °C in some samples, the red coloration intensifies, and discoloration increases, possibly because higher temperatures reduce AB1 adsorption on the photocatalyst. Controlling the ambient temperature was a determining factor in increasing H_2_ production.
- The reaction pH shows minimal variation, likely because the processes taking place generate and consume OH^−^.
- The alkaline environment influences the blank tests: without dye and CuO-TiO_2_, a negative ORP develops over time.
- In the CuO-TiO_2_/NaOH blank tests, an increase in ORP and zero H_2_ production was observed.

**Figure 7:**
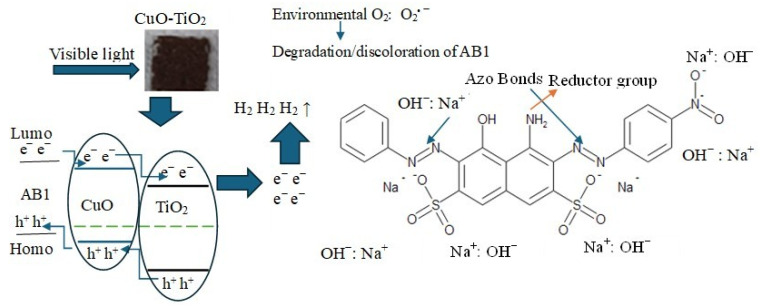
Suggested mechanism of degradation of AB1 dye and evolution of H_2_ in the CuO-TiO_2_/AB1/NaOH system.

The data indicates that as discoloration advances, H_2_ production decreases. Therefore, injecting AB1 into the system becomes necessary to maintain or increase H_2_ production. This process should function as a synergistic system to degrade environmental pollutants like AB1 and produce H_2_, eliminating the need for sacrificial materials [26]. Figure 7 shows a possible route in the simultaneous evolution of H_2_ and decomposition of the AB1 dye. 

## 3. Conclusions

This work demonstrates that the CuO-TiO_2_/AB1/NaOH system with controlled temperature enhances H_2_ generation. CuO-TiO_2_ xerogels exhibited sponge-like structures with anatase crystallite sizes between 19 and 21 nm. Doping of TiO_2_ resulted in a bandgap (Eg) of 2.68 eV, while CuO showed an Eg of 2.01 eV. Photoluminescence measurements demonstrated that CuO-TiO_2_ forms an effective rectifying junction that avoids electron-hole recombination when illuminated. Polarization curves and Bode diagrams showed that the CuO-TiO_2_/AB1/NaOH response is sensitive to NaOH molarity and AB1 concentration conditions, with differences in impedance related to the chemical reactions taking place. Regarding the simultaneous evolution of H_2_ and decomposition of AB1, the synergy between the photocatalyst and the dye occurs if the dye is adsorbed. NaOH does not show evidence of H_2_ being produced. The best degradation was achieved with CuO-TiO_2_/NaOH/AB1 systems under controlled environmental conditions: 0.5 M NaOH and 40 mg/L AB1 at 40 °C with a k of 0.066 min^−1^ and R^2^ of 0.99. In the simultaneous process of H_2_ evolution and AB1 decomposition, it is necessary to decide whether greater H_2_ production or AB1 decomposition is required. However, since AB1 presents challenges with its degradation, this system can aid in its elimination. The highest hydrogen production, 5050 ppb/g_CuO-TiO2_/h, was achieved with the CuO-TiO_2_/NaOH/AB1 system under controlled environmental conditions: 40 °C, 0.5 M NaOH, 40 mg/L AB1, and 6 mL of AB1 injection. The second-best result, 1927 ppb/g_CuO-TiO2_/h, was obtained with the same system but with 8 mL of AB1 injection.

## 4. Materials and Methods

AI use: for the section “Recent Advances in Simultaneous H_2_ Evolution and Contaminant Degradation”, AI online (PopAI, www.popai.pro, Singapore). was used. Articles of interest were uploaded to chatPDF, and a summary of the selected articles was obtained. This summary was then further summarized and formatted to generate the desired text.

### 4.1. Sol-Gel Synthesis of CuO-TiO_2_ Calcined Xerogels

Copper-doped titanium xerogels were synthesized following a procedure previously described in the literature [27]. Briefly, 0.217 g of CuCl_2_ salts (Fermont, Fremont, OH, USA) were dissolved in 50 mL of anhydrous ethylic alcohol (Fermont) acidified with 0.1 mL of HCl (Fermont), with stirring until complete dissolution; the molar ratio of H^+^/Ti = 0.23 was obtained, and EtOH/Ti = 45. Subsequently, 5 mL of 97% titanium isopropoxide (Aldrich, Saint Louis, MO, USA) was added to obtain a Cu/Ti molar ratio of 0.08. After homogenization and agitation (1 min), 2 mL of water (H_2_O/Ti = 4.8) was added to initiate controlled hydrolysis. Stirring continued for 5 min, and the mixture was then allowed to sit until gelation occurred, typically within 20 min to 1 h. The gels were calcined by slowly heating at 1.5 °C/min to 450 °C and maintained at this temperature for one hour. The resulting xerogels were millimetric aggregates with sponge-like structures. Table 4 shows the experimental parameters used for synthesis.

### 4.2. Characterization of CuO-TiO_2_ Calcinated Xerogels

The characterization was carried out by X-ray diffraction (XRD), using a QUANTAX Bruker D2 Phaser diffractometer (35 kV, 20 mA, Yokohama-city, Kanagawa, Japan), with CuKα radiation (λ = 1.548 Å). The crystallite size was calculated from the (101) plane of anatase using the Debye–Scherrer formula. A Hitachi S-5500 Field Emission Scanning Electron Microscope (FE SEM, Tokyo, Japan), equipped with a QUANTAX Bruker 200 Energy Dispersive Spectroscopy (EDS, Yokohama-city, Kanagawa, Japan) detector, was used to determine the composition and morphology. The samples were placed on the surface of an aluminum pin on conductive graphite and analyzed using a 1.5 KeV electron beam. The band gap was calculated using the Kubelka–Munk method, based on transmittance spectra obtained with a Shimadzu UV-31001 PC spectrophotometer, (Mumbai, India). Photoluminescence spectroscopy (PL) was measured with PerkinElmer, LS-55 and LS-45 Fluorescence Spectrometers (E.E.U.U.), using an excitation wavelength of 250 nm.

Polarization curves and electrochemical impedance tests of alkaline dye solutions were performed using a GillAC ACM potentiostat, at various AB1 concentrations (from 40 to 800 mg/L) and NaOH molarities (from 0.3 to 0.5 M NaOH), as summarized in Table 4. The tests were conducted in a three-electrode reactor with thick CuO-TiO_2_ films as the working electrode, graphite as the counter electrode, and silver chloride as the reference electrode. The working electrode consisted of a mixture of CuO-TiO_2_ and diluted transparent varnish (0.4 g of CuO-TiO_2_ and 0.5 mL of varnish) deposited on 2.5 cm × 2.5 cm indium tin oxide (ITO) conductive glass.

### 4.3. H_2_ Evolution and AB1 Degradation Experiments

The experiments were conducted with 100 mL of alkaline AB1 solutions (see Table 1) and working electrodes made of thick CuO-TiO_2_ films. Blank tests were also performed (i.e., alkaline medium without catalyst and dye, or with just one of them). AB1 discoloration was monitored by following the absorption spectrum using a Thermo Scientific Genesys 10S UV-Vis spectrophotometer (E.E.U.U.). The spectrum showed a main peak at 618 nm, which was used to generate the calibration curve and determine the concentration in mg/L. The pH, H_2_ production, and ORP were monitored with a KKMOON Smart pH Meter (Model 10 in 1 Water Quality Tester, China). Measurements were taken every 1 or 4 min, depending on the evolution of H_2_ or the specific test set being conducted.

## Figures and Tables

**Table 1:** Combinations of experiments developed. Systems, CuO-TIO_2_/NaOH, AB1/NaOH, CuO-TiO_2_/AB1/NaOH, and NaOH only.

Experiment	[NaOH] M	[AB1] mg/L	[NaOH] M	[AB1] mg/L	[NaOH] M	[AB1] mg/L
Only NaOH	0.3	---	0.4	---	0.5	---
CuO-TiO_2_/NaOH	0.3	---	0.4	---	0.5	---
AB1/NaOH	0.3	40	0.4	40	0.5	40
AB1/NaOH	0.3	60	0.4	60	0.5	60
AB1/NaOH	0.3	80	0.4	80	0.5	80
CuO-TiO_2_/AB1/NaOH	0.3	40	0.4	40	0.5	40
CuO-TiO_2_/AB1/NaOH	0.3	60	0.4	60	0.5	60
CuO-TiO_2_/AB1/NaOH	0.3	80	0.4	80	0.5	80

**Table 2:** First-order kinetics of some experiments of the CuO-TiO_2_/[AB1]/[NaOH] system.

[AB1]/[NaOH] mg/L/M	T (°C)	k (min^−1^)	R^2^
40	30	0.0459	99
40	30	0.014	0.985
60	30	0.0403	1
80	30	0.009	0.99
40	40	0.055	0.99
40	40	0.066	0.99
60	40	0.0512	0.99

**Table 3:** Injection of AB1 in the CuO-TiO_2_/[AB1]/[NaOH] system for the sustained achievement of H_2_ (Figure 6f).

1st Injection		2nd Injection		3rd Injection		Hydrogen Production
t, min	AB1, mL	t, min	AB1, mL	t, min	AB1, mL	ppb/g_cat_/h
24	2	42	3	51	3	1927
21	3	56	3	---	---	5050

[AB1] = 1000 mg/L.

**Table 4:** Experimental parameters used for synthesis of calcined TiO_2_ and CuO-TiO_2_ xerogels.

Nanomaterial	EtOH/Ti Molar Ratio	H^+^/Ti Molar Ratio	Cu/Ti Molar Ratio	H_2_O/Ti Molar Ratio	Calcined’s Characteristics
Cu-TiO_2_	45	0.23	0.08	4.8	1.5 °C/min to 450 °C for 1 h
TiO_2_	45	0.23	---	4.8	

## Data Availability

The original contributions presented in this study are included in the article. Further inquiries can be directed to the corresponding author.
